# The genome sequence of the Top-horned Hunchback fly, *Acrocera orbiculus *(Fabricius, 1787)

**DOI:** 10.12688/wellcomeopenres.20891.1

**Published:** 2024-02-12

**Authors:** Liam M. Crowley, Neil Phillips, Ed Hardy, Erica McAlister, University of Oxford and Wytham Woods Genome Acquisition Lab, Natural History Museum Genome Acquisition Lab, Darwin Tree of Life Barcoding collective, Wellcome Sanger Institute Tree of Life Management, Samples and Laboratory team, Wellcome Sanger Institute Scientific Operations: Sequencing Operations, Wellcome Sanger Institute Tree of Life Core Informatics team, Tree of Life Core Informatics collective, Darwin Tree of Life Consortium

**Affiliations:** 1 University of Oxford, Oxford, England, UK; 2 Independent researcher, Basildon, England, UK; 3 Independent Researcher, Mickleton, England, UK; 4 Natural History Museum, London, England, UK

**Keywords:** Acrocera orbiculus, Top-horned Hunchback fly, genome sequence, chromosomal, Diptera

## Abstract

We present a genome assembly from an individual female *Acrocera orbiculus* (the Top-horned Hunchback fly; Arthropoda; Insecta; Diptera; Acroceridae). The genome sequence is 221.0 megabases in span. Most of the assembly is scaffolded into 5 chromosomal pseudomolecules, including the X sex chromosome. The mitochondrial genome has also been assembled and is 18.67 kilobases in length. Gene annotation of this assembly on Ensembl identified 10,439 protein coding genes.

## Species taxonomy

Eukaryota; Opisthokonta; Metazoa; Eumetazoa; Bilateria; Protostomia; Ecdysozoa; Panarthropoda; Arthropoda; Mandibulata; Pancrustacea; Hexapoda; Insecta; Dicondylia; Pterygota; Neoptera; Endopterygota; Diptera; Brachycera; Muscomorpha; Nemestrinoidea; Acroceridae; *Acrocera*; *Acrocera orbiculus* (Fabricius, 1787) (NCBI:txid210248).

## Background

*Acrocera orbiculus* (Fabricius, 1787), the Top-horned Hunchback fly, is one of three species of Acroceridae found in the UK, but globally there are around 530 species placed within 55 genera ( Stubbs & Drake, 2014). *Acrocera* is a cosmopolitan genus spanning the Holarctic but most frequently sampled in western Palaearctic ( Kehlmaier & Almeida, 2014).

The adults are small (body length 3–6.56 mm, wing length 3.5–4.5 mm) and are globular, with a shape that is often described as ‘dumpy’. The head is very small and round with the diagnostic features of the genus being that the stylate antennae are positioned on top of the head, the eyes lack hair, and both mouthparts and wing venation are reduced ( Stubbs & Drake, 2014). They have well developed squamae (calypters) and are very good flyers, although not often seen due in some part to the very short emergence period (thought to be only a few days) ( Schlinger, 1987).

*Acrocera orbiculus* are commonly called hunchback flies, small-headed flies or spider-killing flies. The latter name indicates the host preference of their larvae: all *Acrocera* species have larvae which are parasitoids of spiders. The adults congregate for mating and oval eggs are laid on vegetation directly afterwards in clusters of several hundred ( Edwards, 1984).

After three to six weeks and usually at night, the first instar (stage) of the larva emerges. This is a specialised instar called a planidium. It is tiny (0.3–0.4 mm), and actively crawls to search out a host. Upon finding one it attaches itself by its mouthpart to the legs and stays there. Acrocerid larva then undergo hypermetamorphosis, changing into a functionally and morphologically different larval phase as an endoparasite ( Schlinger, 1987). Host records are few, but Kehlmaier and Almeida (2014) list three families within which rearing has been recorded (Amaurobiidae, Clubionidae and Lycosidae). The first instar cuts through the integument and is thought to feed directly on the haemolymph of its host ( Overgaard Nielsen *et al.*, 1999). After a week the first moulting occurs, and it is during this period that the second, more flexible, instar injects itself into the host via the original oral cavity and host wound, leaving the original exuviae as a plug ( Overgaard Nielsen *et al.*, 1999; Toft *et al.*, 2012). The second instar, now an endoparasite, travels through the lymph up through to the abdomen where it resides near the booklungs of the spider, growing in size till the final (fourth) instar. The mature larvae leave the host at this stage to pupate, which results in the death of the spider. Waste material is evacuated from the body pre-pupation and from host to the adult fly emerging has been recorded as just over two weeks ( Kehlmaier & Almeida, 2014).

In the UK this species has a threat status of Least Concern and a rarity status of Nationally Scarce ( Drake, 2017). Data held by the national recording scheme for soldierflies and allies includes records from 42 vice-counties, widely scattered from South Devon northwards to Stirlingshire; it is more frequently seen in the southern parts of its range (and is also known from the Channel Islands) (Martin Harvey, pers. comm.).

Previous molecular analysis of the COI-dataset has been undertaken to help determine whether this species was in fact a complex, as suggested by the variable abdominal pattern found across the range. The findings of Kehlmaier and Almeida (2014) suggest that this is a single species, albeit with a high degree of variation in size, colouration, and some sexual dimorphism.

The genome of the Top-horned Hunchback fly, *Acrocera orbiculus*, was sequenced as part of the Darwin Tree of Life Project, a collaborative effort to sequence all named eukaryotic species in the Atlantic Archipelago of Britain and Ireland.

## Genome sequence report

**Figure 1.**
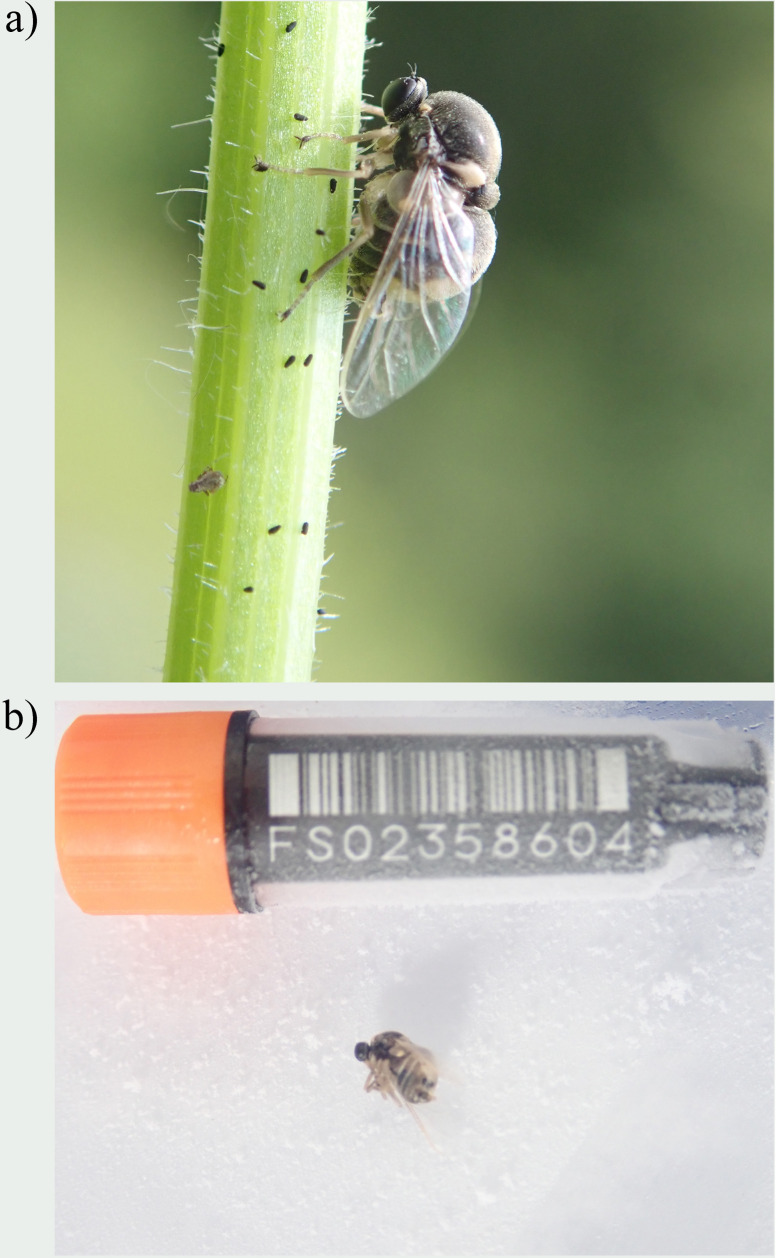
Photographs of the *Acrocera orbiculus* (idAcrOrbc1) specimen used for genome sequencing **a**) Live specimen **b**) Specimen during preservation and processing.

The genome was sequenced from one female *Acrocera orbiculus* ( Figure 1) collected from Wytham Woods, Oxfordshire, UK (51.77, –1.34). A total of 104-fold coverage in Pacific Biosciences single-molecule HiFi long reads was generated. Primary assembly contigs were scaffolded with chromosome conformation Hi-C data. Manual assembly curation corrected 4 missing joins or mis-joins and removed one haplotypic duplication.

**Figure 2.**
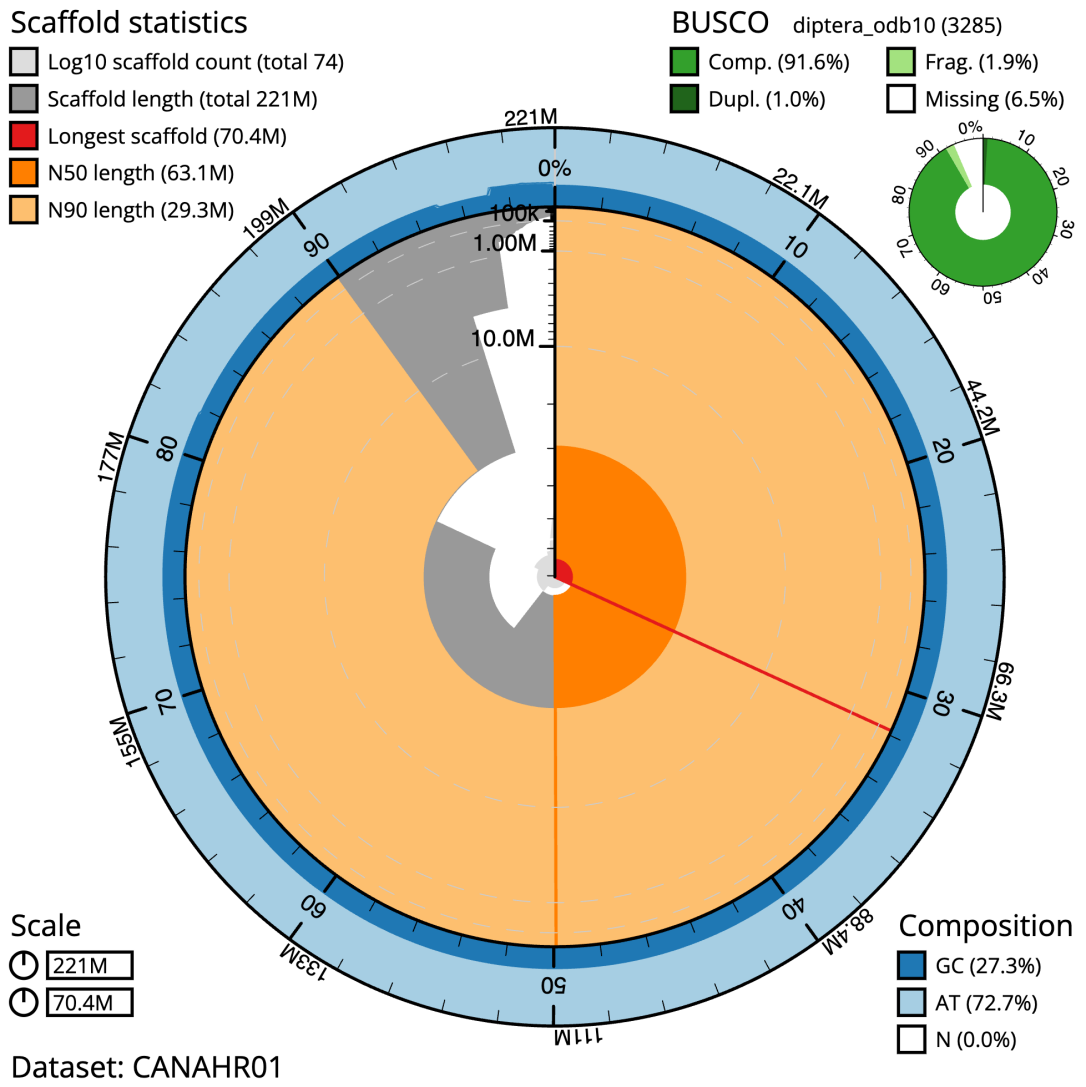
Genome assembly of *Acrocera orbiculus*, idAcrOrbc1.1: metrics. The BlobToolKit Snailplot shows N50 metrics and BUSCO gene completeness. The main plot is divided into 1,000 size-ordered bins around the circumference with each bin representing 0.1% of the 221,032,551 bp assembly. The distribution of scaffold lengths is shown in dark grey with the plot radius scaled to the longest scaffold present in the assembly (70,384,342 bp, shown in red). Orange and pale-orange arcs show the N50 and N90 scaffold lengths (63,123,693 and 29,288,000 bp), respectively. The pale grey spiral shows the cumulative scaffold count on a log scale with white scale lines showing successive orders of magnitude. The blue and pale-blue area around the outside of the plot shows the distribution of GC, AT and N percentages in the same bins as the inner plot. A summary of complete, fragmented, duplicated and missing BUSCO genes in the diptera_odb10 set is shown in the top right. An interactive version of this figure is available at https://blobtoolkit.genomehubs.org/view/CANAHR01/dataset/CANAHR01/snail.

**Figure 3.**
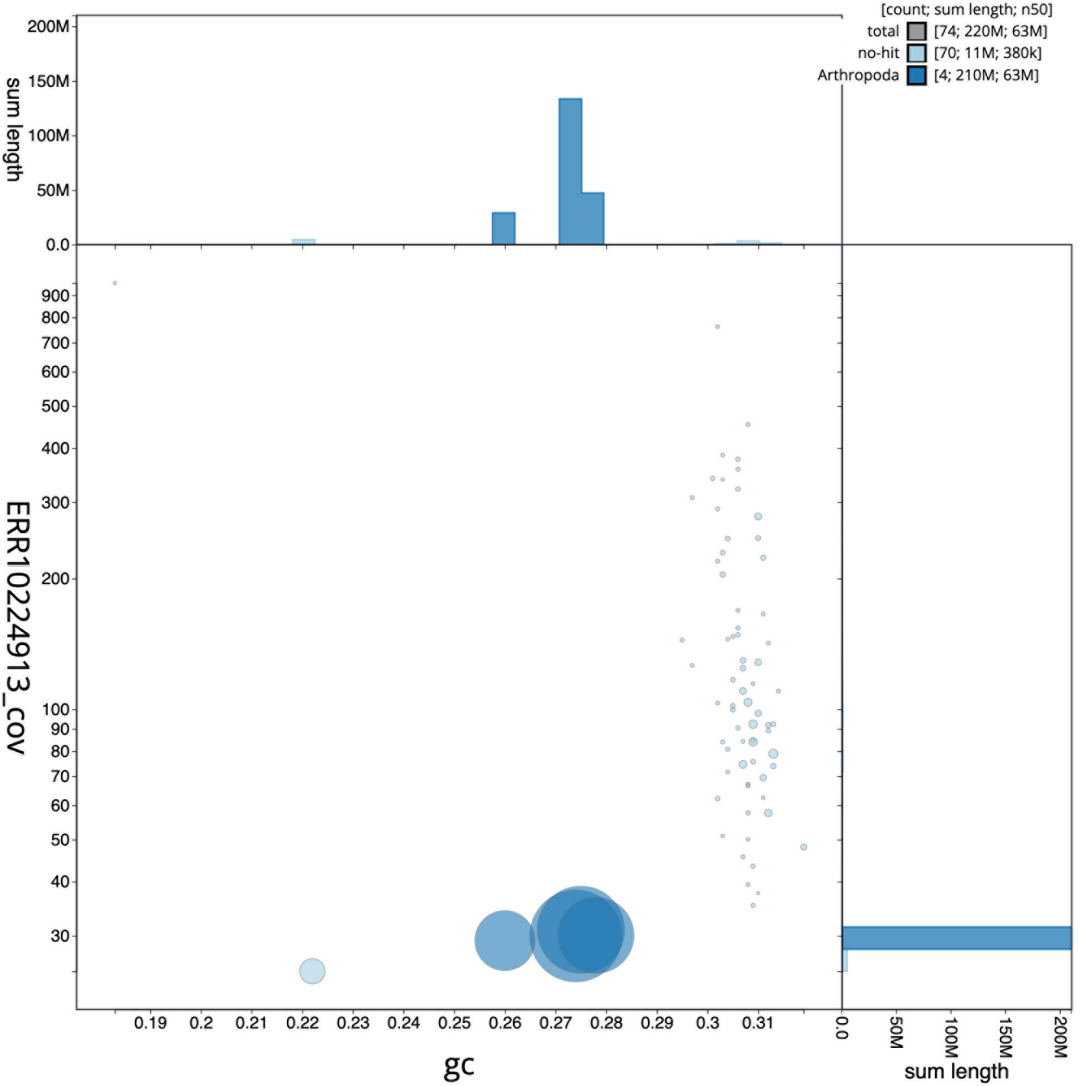
Genome assembly of *Acrocera orbiculus*, idAcrOrbc1.1: BlobToolKit GC-coverage plot. Scaffolds are coloured by phylum. Circles are sized in proportion to scaffold length. Histograms show the distribution of scaffold length sum along each axis. An interactive version of this figure is available at https://blobtoolkit.genomehubs.org/view/CANAHR01/dataset/CANAHR01/blob.

**Figure 4.**
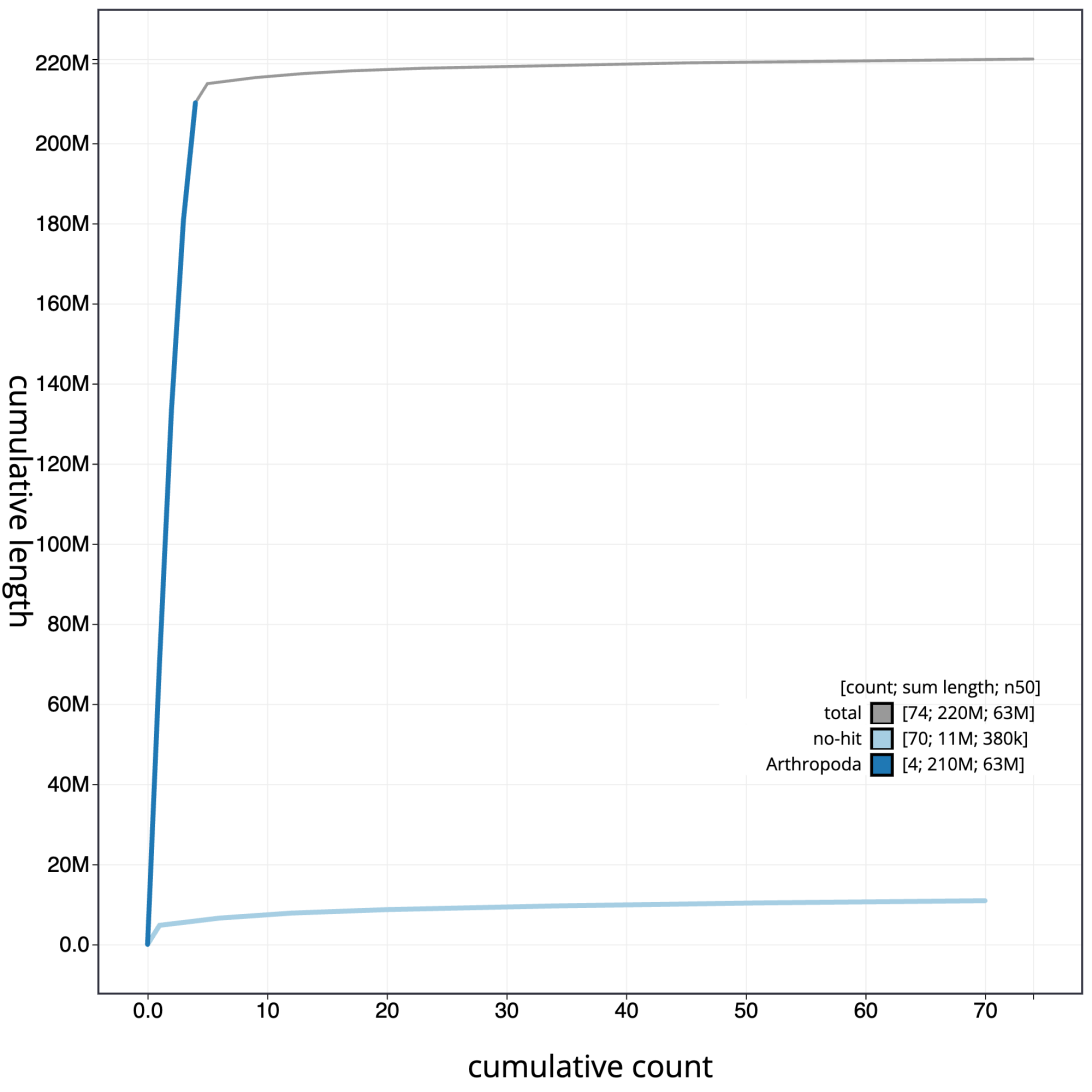
Genome assembly of *Acrocera orbiculus*, idAcrOrbc1.1: BlobToolKit cumulative sequence plot. The grey line shows cumulative length for all scaffolds. Coloured lines show cumulative lengths of scaffolds assigned to each phylum using the buscogenes taxrule. An interactive version of this figure is available at https://blobtoolkit.genomehubs.org/view/CANAHR01/dataset/CANAHR01/cumulative.

**Figure 5.**
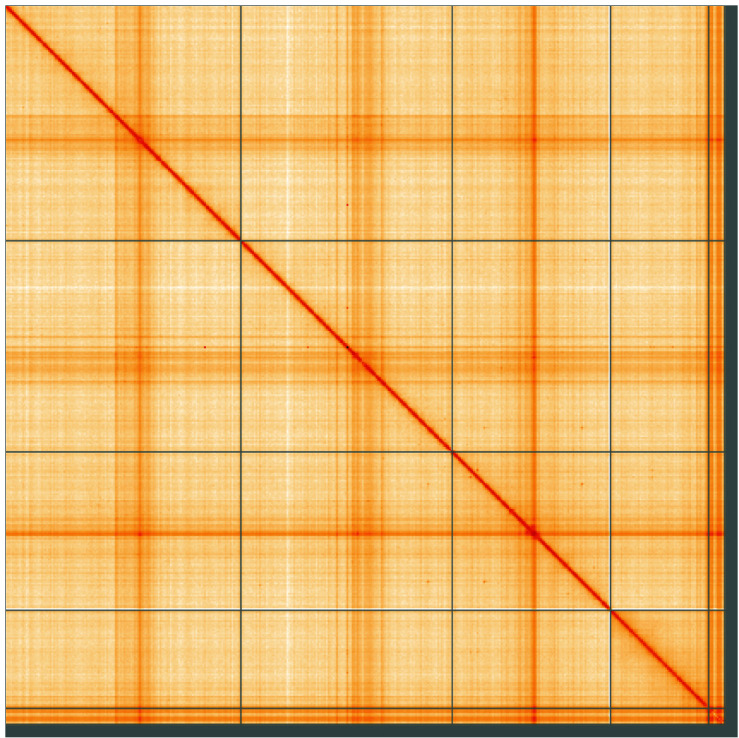
Genome assembly of *Acrocera orbiculus*, idAcrOrbc1.1: Hi-C contact map of the idAcrOrbc1.1 assembly, visualised using HiGlass. Chromosomes are shown in order of size from left to right and top to bottom. An interactive version of this figure may be viewed at https://genome-note-higlass.tol.sanger.ac.uk/l/?d=LcDi2s4OSM-GkorzPMxP3w.

The final assembly has a total length of 221.0 Mb in 73 sequence scaffolds with a scaffold N50 of 63.1 Mb ( Table 1). The snailplot in Figure 2 provides a summary of the assembly statistics, while the distribution of assembly scaffolds on GC proportion and coverage is shown in Figure 3. The cumulative assembly plot in Figure 4 shows curves for subsets of scaffolds assigned to different phyla. Most (99.99%) of the assembly sequence was assigned to 5 chromosomal-level scaffolds, representing 4 autosomes and the X sex chromosome. Chromosome-scale scaffolds confirmed by the Hi-C data are named in order of size ( Figure 5; Table 2). The size of the rRNA clusters on chromosome X is not exact and additional rRNA sequences are in the unlocalised sequences for X. The observed genotype is 4+XX, but the coverage on the repetitive allosome is very unequal. While not fully phased, the assembly deposited is of one haplotype. Contigs corresponding to the second haplotype have also been deposited. The mitochondrial genome was also assembled and can be found as a contig within the multifasta file of the genome submission.

**Table 1.** Genome data for *Acrocera orbiculus*, idAcrOrbc1.1.

Project accession data		
Assembly identifier	idAcrOrbc1.1	
Species	*Acrocera orbiculus*	
Specimen	idAcrOrbc1	
NCBI taxonomy ID	210248	
BioProject	PRJEB55974	
BioSample ID	SAMEA7701562	
Isolate information	idAcrOrbc1	
Assembly metrics *		*Benchmark*
Consensus quality (QV)	65.8	*≥ 50*
*k*-mer completeness	100.0%	*≥ 95%*
BUSCO **	C:91.6%[S:90.7%,D:1.0%], F:1.9%,M:6.5%,n:3,285	*C ≥ 95%*
Percentage of assembly mapped to chromosomes	99.99%	*≥ 95%*
Sex chromosomes	X	*localised homologous pairs*
Organelles	Mitochondrial genome: 18.67 kb	*complete single alleles*
Raw data accessions		
PacificBiosciences SEQUEL II	ERR10224913	
Hi-C Illumina	ERR10297810	
PolyA RNA-Seq Illumina	ERR10378032	
Genome assembly		
Assembly accession	GCA_947359355.1	
*Accession of alternate haplotype*	GCA_947359395.1	
Span (Mb)	221.0	
Number of contigs	76	
Contig N50 length (Mb)	63.1	
Number of scaffolds	73	
Scaffold N50 length (Mb)	63.1	
Longest scaffold (Mb)	70.38	
Genome annotation		
Number of protein-coding genes	10,439	
Number of non-coding genes	1,292	
Number of gene transcripts	19,199	

* Assembly metric benchmarks are adapted from column VGP-2020 of “Table 1: Proposed standards and metrics for defining genome assembly quality” from Rhie *et al.* (2021). ** BUSCO scores based on the diptera_odb10 BUSCO set using version 5.3.2. C = complete [S = single copy, D = duplicated], F = fragmented, M = missing, n = number of orthologues in comparison. A full set of BUSCO scores is available at https://blobtoolkit.genomehubs.org/view/CANAHR01/dataset/CANAHR01/busco.

**Table 2.** Chromosomal pseudomolecules in the genome assembly of *Acrocera orbiculus*, idAcrOrbc1.

INSDC accession	Chromosome	Length (Mb)	GC%
OX375756.1	1	70.38	27.5
OX375757.1	2	63.12	27.5
OX375758.1	3	47.32	28.0
OX375759.1	4	29.29	26.0
OX375760.1	X	4.76	22.0
OX375761.1	MT	0.02	18.5

The estimated Quality Value (QV) of the final assembly is 65.8 with *k*-mer completeness of 100.0%, and the assembly has a BUSCO v5.3.2 completeness of 91.6% (single = 90.7%, duplicated = 1.0%), using the diptera_odb10 reference set ( *n* = 3,285).

Metadata for specimens, barcode results, spectra estimates, sequencing runs, contaminants and pre-curation assembly statistics are given at https://links.tol.sanger.ac.uk/species/210248.

## Genome annotation report

The *Acrocera orbiculus* genome assembly (GCA_947359355.1) was annotated using the Ensembl rapid annotation pipeline ( Table 1; https://rapid.ensembl.org/Acrocera_orbiculus_GCA_947359355.1/Info/Index). The resulting annotation includes 19,199 transcribed mRNAs from 10,439 protein-coding and 1,292 non-coding genes.

## Methods

### Sample acquisition and nucleic acid extraction

A female *Acrocera orbiculus* (specimen ID Ox000701, ToLID idAcrOrbc1), which was used for genome sequencing, was collected from Wytham Woods, Oxfordshire (biological vice-county Berkshire), UK (latitude 51.77, longitude –1.34) on 2020-07-24 by potting. The specimen used for Hi-C sequencing (specimen ID Ox001583, ToLID idAcrOrbc2) was collected from the same location on 2021-07-14. The specimens were collected and identified by Liam Crowley (University of Oxford) and preserved on dry ice.

The specimen used for RNA sequencing (specimen ID NHMUK014561630, ToLID idAcrOrbc3) was collected from South Ockendon, UK (latitude 51.51, longitude 0.29) on 2021-07-25. The specimen was collected by Neil Phillips (Dipterists Forum) and identified by Ed Hardy (Dipterists Forum).

Protocols developed by the Wellcome Sanger Institute (WSI) Tree of Life core laboratory have been deposited on protocols.io ( Denton *et al.*, 2023b). The workflow for high molecular weight (HMW) DNA extraction at the WSI includes a sequence of core procedures: sample preparation; sample homogenisation, DNA extraction, fragmentation, and clean-up. In sample preparation, the idAcrOrbc1 sample was weighed and dissected on dry ice ( Jay *et al.*, 2023). Tissue from the whole organism was homogenised using a PowerMasher II tissue disruptor ( Denton *et al.*, 2023a). HMW DNA was extracted using the Automated MagAttract v1 protocol ( Sheerin *et al.*, 2023). HMW DNA was sheared into an average fragment size of 12–20 kb in a Megaruptor 3 system with speed setting 30 ( Todorovic *et al.*, 2023). Sheared DNA was purified by solid-phase reversible immobilisation ( Strickland *et al.*, 2023): in brief, the method employs a 1.8X ratio of AMPure PB beads to sample to eliminate shorter fragments and concentrate the DNA. The concentration of the sheared and purified DNA was assessed using a Nanodrop spectrophotometer and Qubit Fluorometer and Qubit dsDNA High Sensitivity Assay kit. Fragment size distribution was evaluated by running the sample on the FemtoPulse system.

RNA was extracted from whole organism tissue of idAcrOrbc3 in the Tree of Life Laboratory at the WSI using the RNA Extraction: Automated MagMax™ *mir*Vana protocol ( do Amaral *et al.*, 2023). The RNA concentration was assessed using a Nanodrop spectrophotometer and a Qubit Fluorometer using the Qubit RNA Broad-Range Assay kit. Analysis of the integrity of the RNA was done using the Agilent RNA 6000 Pico Kit and Eukaryotic Total RNA assay.

### Sequencing

Pacific Biosciences HiFi circular consensus DNA sequencing libraries were constructed according to the manufacturers’ instructions. Poly(A) RNA-Seq libraries were constructed using the NEB Ultra II RNA Library Prep kit. DNA and RNA sequencing was performed by the Scientific Operations core at the WSI on Pacific Biosciences SEQUEL II (HiFi) and Illumina NovaSeq 6000 (RNA-Seq) instruments. Hi-C data were also generated from whole organism tissue of idAcrOrbc2 using the Arima2 kit and sequenced on the Illumina NovaSeq 6000 instrument.

### Genome assembly, curation and evaluation

Assembly was carried out with Hifiasm ( Cheng *et al.*, 2021) and haplotypic duplication was identified and removed with purge_dups ( Guan *et al.*, 2020). The assembly was then scaffolded with Hi-C data ( Rao *et al.*, 2014) using YaHS ( Zhou *et al.*, 2023). The assembly was checked for contamination and corrected using the gEVAL system ( Chow *et al.*, 2016) as described previously ( Howe *et al.*, 2021). Manual curation was performed using gEVAL, HiGlass ( Kerpedjiev *et al.*, 2018) and Pretext ( Harry, 2022). The mitochondrial genome was assembled using MitoHiFi ( Uliano-Silva *et al.*, 2023), which runs MitoFinder ( Allio *et al.*, 2020) or MITOS ( Bernt *et al.*, 2013) and uses these annotations to select the final mitochondrial contig and to ensure the general quality of the sequence.

A Hi-C map for the final assembly was produced using bwa-mem2 ( Vasimuddin *et al.*, 2019) in the Cooler file format ( Abdennur & Mirny, 2020). To assess the assembly metrics, the *k*-mer completeness and QV consensus quality values were calculated in Merqury ( Rhie *et al.*, 2020). This work was done using Nextflow ( Di Tommaso *et al.*, 2017) DSL2 pipelines “sanger-tol/readmapping” ( Surana *et al.*, 2023a) and “sanger-tol/genomenote” ( Surana *et al.*, 2023b). The genome was analysed within the BlobToolKit environment ( Challis *et al.*, 2020) and BUSCO scores ( Manni *et al.*, 2021; Simão *et al.*, 2015) were calculated.

Table 3 contains a list of relevant software tool versions and sources.

**Table 3.** Software tools: versions and sources.

Software tool	Version	Source
BlobToolKit	4.1.7	https://github.com/blobtoolkit/blobtoolkit
BUSCO	5.3.2	https://gitlab.com/ezlab/busco
gEVAL	N/A	https://geval.org.uk/
Hifiasm	0.16.1-r375	https://github.com/chhylp123/hifiasm
HiGlass	1.11.6	https://github.com/higlass/higlass
Merqury	MerquryFK	https://github.com/thegenemyers/MERQURY.FK
MitoHiFi	2	https://github.com/marcelauliano/MitoHiFi
PretextView	0.2	https://github.com/wtsi-hpag/PretextView
purge_dups	1.2.3	https://github.com/dfguan/purge_dups
sanger-tol/genomenote	v1.0	https://github.com/sanger-tol/genomenote
sanger-tol/readmapping	1.1.0	https://github.com/sanger-tol/readmapping/tree/1.1.0
YaHS	yahs-1.1.91eebc2	https://github.com/c-zhou/yahs

### Genome annotation

The Ensembl gene annotation system ( Aken *et al.*, 2016) was used to generate annotation for the *Acrocera orbiculus* assembly (GCA_947359355.1). Annotation was created primarily through alignment of transcriptomic data to the genome, with gap filling via protein-to-genome alignments of a select set of proteins from UniProt ( UniProt Consortium, 2019).

### Wellcome Sanger Institute – Legal and Governance

The materials that have contributed to this genome note have been supplied by a Darwin Tree of Life Partner. The submission of materials by a Darwin Tree of Life Partner is subject to the **‘Darwin Tree of Life Project Sampling Code of Practice’**, which can be found in full on the Darwin Tree of Life website here. By agreeing with and signing up to the Sampling Code of Practice, the Darwin Tree of Life Partner agrees they will meet the legal and ethical requirements and standards set out within this document in respect of all samples acquired for, and supplied to, the Darwin Tree of Life Project.

Further, the Wellcome Sanger Institute employs a process whereby due diligence is carried out proportionate to the nature of the materials themselves, and the circumstances under which they have been/are to be collected and provided for use. The purpose of this is to address and mitigate any potential legal and/or ethical implications of receipt and use of the materials as part of the research project, and to ensure that in doing so we align with best practice wherever possible. The overarching areas of consideration are:

•     Ethical review of provenance and sourcing of the material

•     Legality of collection, transfer and use (national and international) 

Each transfer of samples is further undertaken according to a Research Collaboration Agreement or Material Transfer Agreement entered into by the Darwin Tree of Life Partner, Genome Research Limited (operating as the Wellcome Sanger Institute), and in some circumstances other Darwin Tree of Life collaborators.

## Data Availability

European Nucleotide Archive: *Acrocera orbiculus* (top-horned hunchback). Accession number PRJEB55974; https://identifiers.org/ena.embl/PRJEB55974 ( Wellcome Sanger Institute, 2022). The genome sequence is released openly for reuse. The *Acrocera orbiculus* genome sequencing initiative is part of the Darwin Tree of Life (DToL) project. All raw sequence data and the assembly have been deposited in INSDC databases. Raw data and assembly accession identifiers are reported in Table 1.

## Author information

Members of the University of Oxford and Wytham Woods Genome Acquisition Lab are listed here: https://doi.org/10.5281/zenodo.7125292.

Members of the Natural History Museum Genome Acquisition Lab are listed here: https://doi.org/10.5281/zenodo.7139035.

Members of the Darwin Tree of Life Barcoding collective are listed here: https://doi.org/10.5281/zenodo.4893703.

Members of the Wellcome Sanger Institute Tree of Life Management, Samples and Laboratory team are listed here: https://doi.org/10.5281/zenodo.10066175.

Members of Wellcome Sanger Institute Scientific Operations: Sequencing Operations are listed here: https://doi.org/10.5281/zenodo.10043364.

Members of the Wellcome Sanger Institute Tree of Life Core Informatics team are listed here: https://doi.org/10.5281/zenodo.10066637.

Members of the Tree of Life Core Informatics collective are listed here: https://doi.org/10.5281/zenodo.5013541.

Members of the Darwin Tree of Life Consortium are listed here: https://doi.org/10.5281/zenodo.4783558.

## References

[ref-1] Abdennur N Mirny LA : Cooler: Scalable storage for Hi-C data and other genomically labeled arrays. *Bioinformatics.* 2020;36(1):311–316. 10.1093/bioinformatics/btz540 31290943 PMC8205516

[ref-2] Aken BL Ayling S Barrell D : The Ensembl gene annotation system. *Database (Oxford).* 2016;2016: baw093. 10.1093/database/baw093 27337980 PMC4919035

[ref-3] Allio R Schomaker-Bastos A Romiguier J : MitoFinder: Efficient automated large-scale extraction of mitogenomic data in target enrichment phylogenomics. *Mol Ecol Resour.* 2020;20(4):892–905. 10.1111/1755-0998.13160 32243090 PMC7497042

[ref-4] Bernt M Donath A Jühling F : MITOS: Improved *de novo* metazoan mitochondrial genome annotation. *Mol Phylogenet Evol.* 2013;69(2):313–319. 10.1016/j.ympev.2012.08.023 22982435

[ref-5] Challis R Richards E Rajan J : BlobToolKit - interactive quality assessment of genome assemblies. *G3 (Bethesda).* 2020;10(4):1361–1374. 10.1534/g3.119.400908 32071071 PMC7144090

[ref-6] Cheng H Concepcion GT Feng X : Haplotype-resolved *de novo* assembly using phased assembly graphs with hifiasm. *Nat Methods.* 2021;18(2):170–175. 10.1038/s41592-020-01056-5 33526886 PMC7961889

[ref-7] Chow W Brugger K Caccamo M : gEVAL — a web-based browser for evaluating genome assemblies. *Bioinformatics.* 2016;32(16):2508–2510. 10.1093/bioinformatics/btw159 27153597 PMC4978925

[ref-8] Denton A Oatley G Cornwell C : Sanger Tree of Life Sample Homogenisation: PowerMash. *protocols.io.* 2023a. 10.17504/protocols.io.5qpvo3r19v4o/v1

[ref-9] Denton A Yatsenko H Jay J : Sanger Tree of Life Wet Laboratory Protocol Collection V.1. *protocols.io.* 2023b. 10.17504/protocols.io.8epv5xxy6g1b/v1

[ref-10] Di Tommaso P Chatzou M Floden EW : Nextflow enables reproducible computational workflows. *Nat Biotechnol.* 2017;35(4):316–319. 10.1038/nbt.3820 28398311

[ref-11] do Amaral RJV Bates A Denton A : Sanger Tree of Life RNA Extraction: Automated MagMax ^TM^ mirVana. *protocols.io.* 2023. 10.17504/protocols.io.6qpvr36n3vmk/v1

[ref-12] Drake CM : A review of the status of Larger Brachycera flies of Great Britain. Species Status No.29. Natural England Commissioned Reports, Number 192,2017. Reference Source

[ref-13] Edwards M : A further observation of swarming behaviour in Acrocera orbicula (F.) (Dipt., Acroceridae). *The Entomologist’s Monthly Magazine.* 1984;120:236.

[ref-14] Guan D McCarthy SA Wood J : Identifying and removing haplotypic duplication in primary genome assemblies. *Bioinformatics.* 2020;36(9):2896–2898. 10.1093/bioinformatics/btaa025 31971576 PMC7203741

[ref-15] Harry E : PretextView (Paired REad TEXTure Viewer): A desktop application for viewing pretext contact maps. 2022; [Accessed 19 October 2022]. Reference Source

[ref-16] Howe K Chow W Collins J : Significantly improving the quality of genome assemblies through curation. *GigaScience.* Oxford University Press,2021;10(1): giaa153. 10.1093/gigascience/giaa153 33420778 PMC7794651

[ref-17] Jay J Yatsenko H Narváez-Gómez JP : Sanger Tree of Life Sample Preparation: Triage and Dissection. *protocols.io.* 2023. 10.17504/protocols.io.x54v9prmqg3e/v1

[ref-18] Kehlmaier C Almeida JM : New host records for European Acroceridae (Diptera), with discussion of species limits of *Acrocera orbiculus* (Fabricius) based on DNA-barcoding. *Zootaxa.* 2014;3780(1):135–52. 10.11646/zootaxa.3780.1.5 24871830

[ref-19] Kerpedjiev P Abdennur N Lekschas F : HiGlass: web-based visual exploration and analysis of genome interaction maps. *Genome Biol.* 2018;19(1): 125. 10.1186/s13059-018-1486-1 30143029 PMC6109259

[ref-20] Manni M Berkeley MR Seppey M : BUSCO update: Novel and streamlined workflows along with broader and deeper phylogenetic coverage for scoring of eukaryotic, prokaryotic, and viral genomes. *Mol Biol Evol.* 2021;38(10):4647–4654. 10.1093/molbev/msab199 34320186 PMC8476166

[ref-21] Overgaard Nielsen B Funch P Toft S : Self-Injection of a Dipteran Parasitoid into a Spider. *Naturwissenschaften.* 1999;86(11):530–532. 10.1007/s001140050668 10551947

[ref-22] Rao SSP Huntley MH Durand NC : A 3D map of the human genome at kilobase resolution reveals principles of chromatin looping. *Cell.* 2014;159(7):1665–1680. 10.1016/j.cell.2014.11.021 25497547 PMC5635824

[ref-23] Rhie A McCarthy SA Fedrigo O : Towards complete and error-free genome assemblies of all vertebrate species. *Nature.* 2021;592(7856):737–746. 10.1038/s41586-021-03451-0 33911273 PMC8081667

[ref-24] Rhie A Walenz BP Koren S : Merqury: Reference-free quality, completeness, and phasing assessment for genome assemblies. *Genome Biol.* 2020;21(1): 245. 10.1186/s13059-020-02134-9 32928274 PMC7488777

[ref-25] Schlinger EI : The biology of Acroceridae (Diptera): true endoparasitoids of spiders. In: Nentwig, W. (ed.) *Ecophysiology of Spiders.*Berlin: Springer-Verlag,1987;319–327. 10.1007/978-3-642-71552-5

[ref-26] Sheerin E Sampaio F Oatley G : Sanger Tree of Life HMW DNA Extraction: Automated MagAttract v.1. *protocols.io.* 2023. 10.17504/protocols.io.x54v9p2z1g3e/v1

[ref-27] Simão FA Waterhouse RM Ioannidis P : BUSCO: assessing genome assembly and annotation completeness with single-copy orthologs. *Bioinformatics.* 2015;31(19):3210–3212. 10.1093/bioinformatics/btv351 26059717

[ref-28] Strickland M Cornwell C Howard C : Sanger Tree of Life Fragmented DNA clean up: Manual SPRI. *protocols.io.* 2023. 10.17504/protocols.io.kxygx3y1dg8j/v1

[ref-29] Stubbs A Drake M : British Soldierflies and Their Allies.2nd Edition. British Entomological and Natural History Society,2014.

[ref-30] Surana P Muffato M Qi G : sanger-tol/readmapping: sanger-tol/readmapping v1.1.0 - Hebridean Black (1.1.0). *Zenodo.* 2023a. Reference Source

[ref-31] Surana P Muffato M Sadasivan Baby C : sanger-tol/genomenote (v1.0.dev). *Zenodo.* 2023b. Reference Source

[ref-32] Todorovic M Sampaio F Howard C : Sanger Tree of Life HMW DNA Fragmentation: Diagenode Megaruptor®3 for PacBio HiFi. *protocols.io.* 2023. 10.17504/protocols.io.8epv5x2zjg1b/v1

[ref-33] Toft S Overgaard Nielsen B Funch P : Parasitoid suppression and life-history modifications in a wolf spider following infection by larvae of an acrocerid fly. *The J of Arachnology.* 2012;40(1):13–17. 10.1636/P11-28.1

[ref-34] Uliano-Silva M Ferreira JGRN Krasheninnikova K : MitoHiFi: a python pipeline for mitochondrial genome assembly from PacBio high fidelity reads. *BMC Bioinformatics.* 2023;24(1): 288. 10.1186/s12859-023-05385-y 37464285 PMC10354987

[ref-35] UniProt Consortium: UniProt: a worldwide hub of protein knowledge. *Nucleic Acids Res.* 2019;47(D1):D506–D515. 10.1093/nar/gky1049 30395287 PMC6323992

[ref-36] Vasimuddin Md Misra S Li H : Efficient Architecture-Aware Acceleration of BWA-MEM for Multicore Systems.In: *2019 IEEE International Parallel and Distributed Processing Symposium (IPDPS).*IEEE,2019;314–324. 10.1109/IPDPS.2019.00041

[ref-38] Wellcome Sanger Institute: The genome sequence of the Top-horned Hunchback fly, *Acrocera orbiculus* (Fabricius, 1787). European Nucleotide Archive.[dataset], accession number PRJEB55974,2022.

[ref-37] Zhou C McCarthy SA Durbin R : YaHS: yet another Hi-C scaffolding tool. *Bioinformatics.* 2023;39(1): btac808. 10.1093/bioinformatics/btac808 36525368 PMC9848053

