# Supplementary material for: The genome sequence of the Top-horned Hunchback fly, Acrocera orbiculus (Fabricius, 1787)
Source: Wellcome Open Res. 2024 Feb 12;9:25. [Version 1] doi: 10.12688/wellcomeopenres.20891.1 (PMC12344402; doi:10.12688/wellcomeopenres.20891.1)
Supplement: Peer review history

# Reviewer report: Reviewer response for version 1

**DOI:** 10.21956/wellcomeopenres.23117.r127907

**Published:** 2025-08-12

**Authors:** Anthony Bayega

**Affiliations:** 1 McGill Genome Centre, Department of Human Genetics, Canada's Michael Smith Genome Sciences Centre, Vancouver, British Columbia, Canada

Crowley al. provide a high quality chromosomal-level genome assembly of the top-horned Hunchback fly, *Acrocera orbiculus*, a commendable effort. My comments follow below:

 1. In Figure 3, the label on the Y-axis which reads “ERR1…” could be changed to something more informative to help the reader make sense of this figure.

 Overall, the authors provide a good-quality genome and also assign an astonishing 99.99% of it to chromosomes. Although much work remains to order the scaffolds, fully phase the contigs and scaffolds, and complete the gaps and also structurally and functionally annotate the genome, the current work will indeed be valuable to the whole community.

Are sufficient details of methods and materials provided to allow replication by others?

Yes

Is the rationale for creating the dataset(s) clearly described?

Yes

Are the datasets clearly presented in a useable and accessible format?

Yes

Are the protocols appropriate and is the work technically sound?

Yes

Reviewer Expertise:

Genomics

I confirm that I have read this submission and believe that I have an appropriate level of expertise to confirm that it is of an acceptable scientific standard.

# Reviewer report: Reviewer response for version 1

**DOI:** 10.21956/wellcomeopenres.23117.r81900

**Published:** 2024-05-08

**Authors:** Darren Obbard

**Affiliations:** 1 The University of Edinburgh, Edinburgh, Scotland, UK

This data note reports the sequencing and assembly of the genome of Acrocera orbiculus as part of the “Darwin Tree of Life” programme. In common with other data notes from this research effort, the reporting is standardised and quite brief. As such, I have very few comments to make.

 The approach is state-of-the-art, the raw data appear to be of a suitably high quality, and the assembly methods are appropriate. The public availability of raw data and genome assembly are appropriate. The resulting genome is likely to be of very high quality, and I have no doubt that it will be of great value to any researchers working on this fascinating group of flies, or on the comparative or evolutionary genomics of insects more generally.

 At this point, I usually make small suggestions for improving the introductory text – but on this occasion I really don’t have any. It is comprehensive, engaging and well written—the very model of what I would hope for in a ‘genome note’.

 I have one possible typo:

- The methods state: “A female Acrocera orbiculus … was collected from Wytham Woods, Oxfordshire … by potting”. It is possible that ‘potting’ is a standard collection technique that I am unaware of, in which case a brief description is warranted. On the other hand, ‘by pooting’ seems pretty likely (i.e. ‘aspirated’ with a ‘pooter’, after the eponymous Frederick William Poos, Jr.).

And one suggestion for improvement

- Please make the photographic figure, and especially the fly image bigger. Maybe also find a picture of a male to present any contrast and a possible host species to enrich the natural history. Have any of the hosts been sequenced?

Instead, and unusually, I have a question about the assembly and annotation.

- Given the apparent completeness of the assembly, it seems very surprising that BUSCO completeness is only 91%, and that the Ensembl rapid pipeline can only find 10,439 protein coding genes. Do we think this reflects real biology (gene loss in this lineage), or is it an annotation artefact? In particular, could it be an artefact of high divergence from references, or could it be caused by the relatively high AT content of the genome? (only 22% GC on the X). Or was the RNA quality/quantity too low (very small sample!). I think this should be commented on in the manuscript.

Are sufficient details of methods and materials provided to allow replication by others?

Yes

Is the rationale for creating the dataset(s) clearly described?

Yes

Are the datasets clearly presented in a useable and accessible format?

Yes

Are the protocols appropriate and is the work technically sound?

Yes

Reviewer Expertise:

Evolutionary genetics and genomics of invertebrates and their parasites, particularly Drosophilidae and their viruses.

I confirm that I have read this submission and believe that I have an appropriate level of expertise to confirm that it is of an acceptable scientific standard.
